# Blood-Derived Inflammatory Indices Across Essential Tremor, Parkinson’s Disease, and Progressive Supranuclear Palsy: An Exploratory Retrospective Analysis

**DOI:** 10.3390/diseases14080281

**Published:** 2026-08-05

**Authors:** Aleksandra Hejnosz, Bartosz Migda, Natalia Madetko-Alster, Dagmara Otto-Ślusarczyk, Piotr Alster

**Affiliations:** 1 Department of Neurology, Medical University of Warsaw, 03-242 Warsaw, Poland; 2 Diagnostic Ultrasound Lab, Department of Pediatric Radiology, Medical University of Warsaw, 02-091 Warsaw, Poland; 3 Department of Biochemistry, Medical University of Warsaw, 02-097 Warsaw, Poland

**Keywords:** essential tremor, Parkinson’s disease, progressive supranuclear palsy, inflammatory biomarkers, neuroinflammation

## Abstract

**Background/Objectives**: Evidence suggests that inflammation contributes to the pathogenesis of neurodegenerative disorders. The utility of blood-derived inflammatory biomarkers in differentiating neurodegenerative disorders remains incompletely understood. The aim of this study was to compare peripheral inflammatory markers in patients with essential tremor (ET), Parkinson’s disease (PD), progressive supranuclear palsy (PSP), and control participants. **Methods**: This retrospective study included 44 patients with ET, 47 with PD, 44 with PSP, and 45 control participants. The neutrophil-to-lymphocyte ratio (NLR), platelet-to-lymphocyte ratio (PLR), monocyte-to-lymphocyte ratio (MLR), systemic immune-inflammation index (SII), systemic inflammation response index (SIRI), and aggregate index of systemic inflammation (AISI) were calculated from routine complete blood counts. Between-group comparisons were performed using the Kruskal–Wallis test, with a Holm correction across the six indices. The effect sizes were also estimated. **Results**: The PLR was the only inflammatory marker that significantly differentiated the analyzed groups (*p* = 0.045), with the highest value observed in PSP patients and the lowest in ET patients. A post hoc analysis indicated different PLR values in PSP than in ET patients (Cliff’s delta = 0.336, small-to-moderate effect). However, the overall association did not remain statistically significant after Holm correction across the six indices (adjusted *p* = 0.268), and the diagnostic group was not independently associated with PLR after adjustment for age and sex. No statistically significant differences were observed for the NLR, MLR, SII, SIRI, or AISI. Nevertheless, PSP patients consistently exhibited the highest median values of the NLR, SII, and AISI, whereas ET patients generally showed lower inflammatory marker levels. **Conclusions**: PLR was the only inflammatory marker that significantly differentiated the analyzed groups and was the highest among patients with PSP. The observed trends suggest a tendency toward greater peripheral immune activation in PSP compared with PD and ET. Larger prospective studies incorporating both inflammatory and neurodegenerative biomarkers are warranted to validate these findings.

## 1. Introduction

Current evidence suggests that neuroinflammatory mechanisms play a significant role in the development and progression of neurodegenerative disorders. Neuroinflammatory processes have been widely implicated in several neurodegenerative diseases, including Parkinson’s disease (PD), progressive supranuclear palsy (PSP), Alzheimer’s disease, and essential tremor (ET).

In PD, neuroinflammation has been linked to dopaminergic neuronal loss and α-synuclein aggregation [1]. Activated microglia may contribute to neuronal injury through the production of inflammatory mediators, the induction of oxidative stress, and the amplification of α-synuclein-induced immune responses [2]. However, the precise mechanisms underlying the bidirectional relationship between neuroinflammation and neurodegeneration remain incompletely understood.

Similarly, growing evidence suggests that inflammatory pathways may contribute to the pathophysiology of ET. Neuroinflammatory changes have been proposed to affect cerebellar networks and cerebello-thalamo-cortical circuits [3,4], although the exact role of inflammation in disease initiation and progression remains unclear. In PSP, a primary tauopathy [5], activated microglia [6] and elevated inflammatory mediators, including increased IL-1β expression in the substantia nigra [7], have been reported, suggesting that neuroinflammation may contribute to tau accumulation, neuronal dysfunction, and disease progression.

Increasing attention has also been directed toward peripheral inflammation as a potential reflection of ongoing neurodegenerative processes. Peripheral blood-derived inflammatory markers have emerged as accessible and cost-effective biomarkers of systemic inflammatory activity. Given the growing evidence linking systemic inflammation with neurodegeneration, further investigation of peripheral inflammatory markers may improve our understanding of disease-specific inflammatory profiles and potentially contribute to the development of novel diagnostic and prognostic tools.

The NLR was initially described as an accessible measure of systemic inflammation in oncological intensive care unit patients [8]. The SII was subsequently developed using neutrophil, platelet, and lymphocyte counts and showed prognostic significance in hepatocellular carcinoma [9], whereas the SIRI, incorporating neutrophils, monocytes, and lymphocytes, was introduced as a prognostic index in pancreatic cancer [10]. The AISI further combined neutrophil, monocyte, platelet, and lymphocyte count and was evaluated in surgical patients and subsequently in idiopathic pulmonary fibrosis [11,12].

Panels that include NLR, PLR, MLR, SII, and SIRI have been investigated in glioblastoma [13], while a recent longitudinal analysis of Parkinson’s Progression Markers Initiative data evaluated the same six indices used in the present study: the NLR, PLR, MLR, SII, SIRI, and AISI in patients with PD [14]. Previous studies have also assessed the NLR and PLR in ET [15,16] and PSP patients [17,18]. However, these studies generally evaluated individual disorders and direct comparisons of the same extended panel across ET, PD, and PSP remain scarce. We therefore examined whether these readily available indices show different distributions across the three disorders within a single cohort. Because evidence regarding the SIRI and AISI in ET and PSP patients remains limited, their inclusion was exploratory and was not based on an assumption of established diagnostic validity. Although peripheral inflammation has been reported in ET, PD and PSP [17,19,20], whether the inflammatory profiles differ between these disorders remains incompletely understood.

Therefore, the aim of the present study was to evaluate selected peripheral inflammatory indices in patients with ET, Parkinson’s disease, or PSP.

## 2. Materials and Methods

### 2.1. Study Design and Participants

This retrospective cross-sectional study included patients with a clinical diagnosis of ET, PD, or PSP who were either hospitalized at the Department of Neurology or evaluated at the Movement Disorders Outpatient Clinic of the Medical University of Warsaw, Poland. Patients with PD were diagnosed according to the Movement Disorder Society diagnostic criteria for Parkinson’s disease [21]. Patients with PSP were diagnosed according to the Movement Disorder Society criteria for PSP [22]. Patients with ET were diagnosed according to the International Parkinson and Movement Disorder Society consensus statement on the classification of tremors [23]. The PSP phenotypes were not analyzed separately due to the limited sample size.

Control participants were recruited from individuals who underwent routine laboratory testing at the Department of Neurology and the affiliated Movement Disorders Outpatient Clinic of the Medical University of Warsaw and who had no history of neurodegenerative, autoimmune, inflammatory, infectious, hematological, or malignant disorders.

All of the participants were screened for conditions that could influence peripheral inflammatory status. The exclusion criteria included active or chronic infection, autoimmune or inflammatory disease, malignancies, hematological disorders, immunosuppressive treatment, significant cardiovascular or cerebrovascular disease, diabetes mellitus, documented and clinically relevant vascular brain lesions when available in medical records or neuroimaging reports, a leukocyte count > 11,000 cells/μL, and any other neurological disorder potentially affecting the immune profile. Individuals with incomplete clinical or laboratory data were also excluded.

This study was conducted in accordance with the Declaration of Helsinki and approved by the Bioethics Committee of the Medical University of Warsaw, Poland (protocol code: AKBE/146/2026; date of approval: 18 May 2026).

### 2.2. Data Collection

Demographic and clinical data, including age, sex, diagnosis, disease duration, and current pharmacological treatment, were obtained through a review of medical records. Laboratory data were collected from routine peripheral blood examinations performed during hospitalization or a visit to the clinic. Blood samples were obtained as part of routine clinical assessment at the time of hospitalization or outpatient evaluation and were analyzed in the central hospital laboratory. If multiple laboratory assessments were available, only the first available measurement was included in the analysis. The complete blood count parameters were measured using an automated hematology analyzer (Sysmex XS-1000i, Sysmex Corporation, Kobe, Japan).

The following hematological parameters were recorded: the total white blood cell count, neutrophil count, lymphocyte count, monocyte count, and platelet count.

### 2.3. Calculation of Inflammatory Indices

The hematological indices were calculated as follows, using absolute blood cell counts obtained from the same complete blood count. Neutrophil-to-lymphocyte ratio (NLR) = neutrophil count/lymphocyte count [8]. Platelet-to-lymphocyte ratio (PLR) = platelet count/lymphocyte count [12]. Monocyte-to-lymphocyte ratio (MLR) = monocyte count/lymphocyte count [12]. Systemic immune-inflammation index (SII) = (platelet count × neutrophil count)/lymphocyte count [9]. Systemic inflammation response index (SIRI) = (neutrophil count × monocyte count)/lymphocyte count [10]. Aggregate index of systemic inflammation (AISI) = (neutrophil count × platelet count × monocyte count)/lymphocyte count [11,12].

### 2.4. Statistical Analysis

Statistical analyses were performed using the Statistica software (Statistica 13.1, TIBCO Software Inc., Tulsa, Oklahoma, United States.). Continuous variables were presented as the mean ± standard deviation (SD) for normally distributed data or as the median and interquartile range (IQR) for non-normally distributed variables. Categorical variables were expressed as counts and percentages. The normality of data distribution was assessed using the Shapiro–Wilk test. As the majority of the analyzed variables did not follow a normal distribution, non-parametric statistical methods were applied. Quantitative parameters were compared among the four groups (ET, PD, PSP, and control participants) using the Kruskal–Wallis test. When statistically significant differences were detected, post hoc pairwise comparisons were performed using Dunn’s test with a correction for multiple comparisons. To account for multiple testing across six primary inflammatory markers, the *p*-values obtained from the Kruskal–Wallis tests were additionally adjusted using the Holm procedure. Effect size for the Kruskal–Wallis test was estimated using epsilon-squared (ε^2^), whereas Cliff’s delta was calculated for the post hoc comparison between PSP and ET patients. Furthermore, a multivariable linear regression analysis using the log-transformed PLR as the dependent variable and adjusted for age and sex was performed to assess the potential influence of confounding factors. As a sensitivity analysis addressing the potential influence of clinically detectable systemic inflammation, all six inflammatory indices were compared again among the four study groups in participants with an available CRP measurement and a CRP concentration ≤ 5 mg/L. The same Kruskal–Wallis procedure and effect-size estimation as in the primary analysis were applied. A *p*-value < 0.05 was considered statistically significant.

## 3. Results

### 3.1. Study Population

The study population comprised a total of 180 participants. The largest group consisted of patients with PD (*n* = 47), while the ET and PSP groups each included *n* = 44 patients. The control group consisted of *n* = 45 individuals. The highest mean age was observed in the PSP group (67.3 ± 6.4 years), whereas patients with ET constituted the youngest group (61.6 ± 14.3 years). The PD and control groups had similar mean ages of 63.6 ± 10.7 and 63.3 ± 11.0 years, respectively. Men predominated in the ET group (54.5%), whereas women slightly outnumbered men in the remaining groups. The highest proportion of female participants was observed in the control group (57.8%), followed by the PSP group (56.8%). Detailed demographic characteristics are presented in Table 1.

### 3.2. Comparison of Peripheral Inflammatory Markers

The results of the comparative analysis of inflammatory indices among the ET, PD, PSP, and control groups are presented in Table 2. The distributions of the inflammatory indices are additionally presented in Supplementary Figures S1–S6, whereas the absolute neutrophil, lymphocyte, monocyte, and platelet counts are shown in Supplementary Table S1.

Among all the analyzed inflammatory markers, the PLR showed the lowest nominal *p*-value (*p* = 0.045). The highest PLR values were observed in patients with PSP (median 142.93, IQR 112.17–183.75), whereas the lowest values were found in the ET group (median 112.03, IQR 88.29–141.37). The corresponding effect size was small (ε^2^ = 0.029; Supplementary Table S2). The post hoc analysis demonstrated a difference in PLR values between PSP and ET patients (Cliff’s delta = 0.336, small-to-moderate effect). However, the overall association did not remain statistically significant after the Holm correction for multiple testing (adjusted *p* = 0.268; Supplementary Table S3). In the multivariable linear regression analysis adjusted for age and sex, the diagnostic group was not independently associated with the PLR. No statistically significant differences were observed for the remaining inflammatory indices, including the NLR (*p* = 0.524), MLR (*p* = 0.898), SII (*p* = 0.131), SIRI (*p* = 0.675), and AISI (*p* = 0.168). Nevertheless, patients with PSP exhibited the highest median NLR, SII, and AISI values among all the study groups, whereas lower values of the inflammatory indices were generally observed in ET patients.

A sensitivity analysis was performed in participants with available CRP concentrations ≤ 5 mg/L (ET, *n* = 19; PD, *n* = 35; PSP, *n* = 23; and control participants, *n* = 24). None of the six inflammatory indices differed significantly among the groups. For PLR, the between-group difference was not significant (H = 3.612, *p* = 0.307, ε^2^ = 0.006). The results of the sensitivity analysis are presented in Supplementary Table S4.

## 4. Discussion

Although most peripheral inflammatory indices did not differ significantly between the analyzed groups, several potentially relevant numerical trends were observed. Patients with PSP showed the highest median NLR, PLR, SII, and AISI values, whereas patients with ET generally had lower inflammatory marker levels. The PLR was the only marker that significantly differentiated the groups, with the highest values observed in PSP patients and the lowest in ET. The post hoc analysis confirmed that PLR was significantly higher in PSP than in ET patients. These findings may suggest that platelet-related inflammatory mechanisms are more pronounced in PSP than in ET; however, this interpretation requires caution because the remaining inflammatory indices did not reach statistical significance.

Platelets are increasingly being recognized not only as mediators of hemostasis and thrombosis but also as active regulators of inflammation and immune responses. Following activation, platelets interact with leukocytes and endothelial cells through molecules such as P-selectin and the CD40 ligand and release cytokines, chemokines, damage-associated molecular patterns, and extracellular vesicles. These processes can promote platelet–leukocyte aggregate formation, leukocyte recruitment, endothelial activation, and platelet–neutrophil interactions, leading to neutrophil extracellular trap formation and thromboinflammation [24,25]. In experimental neuroinflammatory settings, activated platelets have also been implicated in blood–brain barrier dysfunction and glial activation [26,27,28]. In this context, the PLR may be considered a readily accessible count-based index influenced by platelet and lymphocyte numbers.

The potential involvement of platelets in neurodegeneration may also relate to the presence and processing of neurodegeneration-associated proteins, although the strength and disease specificity of the evidence differs. Human platelets contain full-length amyloid precursor protein (APP) and APP-derived fragments. Platelet stimulation induces APP processing and the release of soluble APP species [29,30]. APP770 is highly enriched in platelets among peripheral blood cells, and soluble APP770 release is correlated with soluble CD40 ligand, supporting its relationship with platelet activation [31]. Earlier work reported the release of preformed amyloid-β (Aβ) from activated platelets, whereas Miura et al. did not detect Aβ40/42 under their experimental conditions, suggesting that the detected products may depend on the agonist and assay used [30,31]. In experimental Alzheimer’s disease and cerebral amyloid angiopathy models, Aβ40 can activate platelet integrin αIIbβ3 and induce ADP and clusterin release, thereby promoting vascular Aβ aggregation [32]. Tau is likewise detectable in human platelets, including in heterocomplexes with Aβ and α-synuclein [33]. Small exploratory studies in Alzheimer’s disease associated platelet tau patterns with cognitive impairment or regional brain atrophy [34,35], whereas another study found that platelet tau measures did not discriminate patients with Alzheimer’s disease from controls [36]. Thus, the evidence is strongest for APP/Aβ-related platelet biology, whereas the biological and biomarker significance of platelet tau remains uncertain.

The higher PLR observed in PSP patients may reflect a more pronounced systemic inflammatory response associated with greater neurodegenerative and systemic disease burden. PSP is a four-repeat tauopathy [5] characterized by rapid disability progression and relatively short survival [37], and evidence concerning systemic inflammatory alterations remains limited and heterogeneous. Previous studies have assessed blood-cell-derived inflammatory indices, circulating cytokines, peripheral immune-cell populations, selected inflammatory markers in serum and cerebrospinal fluid, and neuroimaging measures of microglial activation [37,38]. Conversely, the relatively low inflammatory indices observed in ET may be consistent with the view that inflammatory alterations in ET are subtle, heterogeneous, and possibly phenotype-dependent [19,39]. This interpretation is also consistent with previous ET studies, although the available evidence remains inconsistent.

Tak and Sengül found no statistically significant differences in the NLR or PLR between 67 patients with ET and 40 healthy controls, despite numerically higher PLR values in ET patients [15]. Their study included a relatively young population, with mean ages of approximately 25 years in the ET group and 27 years in the control group. In contrast, Aygün and Dundar analyzed 103 patients with ET and 103 age- and sex-matched healthy controls, whose mean ages were approximately 45 and 47 years, respectively. They reported higher leukocyte counts, C-reactive protein (CRP) levels, and leukocyte-based inflammatory indices, including the NLR, PLR, SII, and SIRI, in patients with ET. However, the CRP and SIRI, rather than the PLR, were independent predictors of ET, and the SIRI showed the best diagnostic performance and correlated positively with tremor severity [16]. Differences in the sample size, age distribution, study design, and hematological components underlying the calculated indices may therefore partly explain the discrepant findings.

Previous studies in parkinsonian syndromes provide partial support for the relevance of the PLR and related inflammatory indices. Madetko et al. reported significantly higher NLR and PLR values in patients with PD compared with healthy controls [40]. In the same study, patients with multiple system atrophy, parkinsonian type (MSA-P) showed an increased NLR, but not PLR, compared with controls, while no significant differences in the NLR or PLR were observed between PD and MSA-P patients. These findings suggest that different parkinsonian syndromes may present partially distinct peripheral inflammatory patterns. Another study showed that higher PLR, granulocyte-to-lymphocyte ratio, and adapted SII were associated with prevalent PD, although these associations were largely explained by the lymphocyte count and were not associated with the incident PD risk [20]. Therefore, the PLR should be interpreted as a nonspecific and component-dependent marker rather than as a disease-specific inflammatory biomarker.

This issue is particularly important because ratio-based inflammatory indices may reflect different hematological mechanisms across disorders. Changes in the PLR, NLR, SII, or AISI may result from increased platelet, neutrophil, or monocyte counts, but also from reduced lymphocyte counts. Dommershuijsen et al. showed in PD patients that associations between several inflammatory ratios and prevalent disease were largely explained by the lymphocyte count [20]. In contrast, Muñoz-Delgado et al. reported higher NLR values in both PSP and PD patients compared with healthy controls, with no significant difference between the PSP and PD patients [18]. Importantly, the altered immune profile appeared to differ between disorders: in PSP patients, an increased NLR was mainly related to higher neutrophil counts, whereas in PD patients it was more closely associated with lower lymphocyte counts; lymphocyte count did not differ between PSP patients and controls [18]. These findings suggest that PSP may have a more innate or myeloid-driven peripheral immune profile, although they do not directly explain the mechanism underlying the PLR. In the present study, the PSP patients showed the highest median values of the PLR, SII, and AISI, which may indicate a broader peripheral inflammatory profile. To facilitate the interpretation of this finding, the absolute neutrophil, lymphocyte, monocyte, and platelet counts are provided in Supplementary Table S1. Nevertheless, because the PLR is a composite index, it remains uncertain whether the observed differences were driven primarily by the platelet count, lymphocyte count, or both. Although evidence regarding the PLR in PSP patients remains limited, several studies support its potential relevance. Madetko-Alster et al. evaluated the NLR and PLR in patients with progressive supranuclear palsy-Richardson syndrome (PSP-RS) and progressive supranuclear palsy-parkinsonism and analyzed their associations with the serum and cerebrospinal fluid (CSF) concentrations of IL-1β and IL-6. They found a significant positive correlation between serum IL-6 and the PLR in PSP-RS, whereas no significant correlations were observed between the NLR/PLR and the CSF interleukin levels [17]. These findings suggest that the PLR may partly reflect serum cytokine-related peripheral inflammatory activity in selected PSP phenotypes, although this observation requires confirmation in larger cohorts. The general hematological evidence also supports a possible relationship between systemic inflammation and platelet-related indices, as IL-6 has been shown to stimulate thrombopoiesis through thrombopoietin and to contribute to inflammatory thrombocytosis [41]. In addition, Tang et al. described the relationship between inflammatory pathways, Toll-like receptors, megakaryocyte differentiation, and platelet production [42]. These mechanisms are not specific to PSP, but they provide biological support for a possible link between systemic inflammation and platelet-related indices.

Further support for the relevance of inflammatory indices in PSP comes from studies relating them to clinical and neuroimaging features. Chunowski et al. reported that both the NLR and PLR were negatively correlated with Montreal Cognitive Assessment scores in PSP patients, suggesting that higher inflammatory indices may be associated with worse cognitive performance [43]. This does not prove causality, but it supports the interpretation that inflammatory indices may be linked to disease burden. In a recent pilot study, Alster et al. reported higher platelet counts and higher PLR values in patients with PSP compared with healthy controls, although the PLR difference was borderline [44]. This finding is consistent with the direction of the present observations, although the association observed in our cohort did not remain statistically significant after correction for multiple testing. Moreover, in the same study, the NLR was lower in PSP patients than in controls, indicating that peripheral inflammatory indices may vary across cohorts depending on the distribution of individual blood cell populations, demographic factors, and methodological differences [44].

The pattern observed in the present study also appears to be broadly consistent with previously reported differences in neurofilament light chain (NfL) concentrations across movement disorders. NfL is a well-established marker of neuroaxonal injury [45,46] and has consistently been reported at higher concentrations in atypical parkinsonian syndromes, including PSP, than in PD, supporting its utility as a biomarker of more extensive neurodegeneration [47,48]. Serum NfL concentrations were also shown to be significantly higher in PD patients than in ET patients and control participants and were associated with motor and cognitive impairment [49,50]. Although NfL was not assessed in the present study, the distribution of inflammatory indices showed a similar direction, with the highest values generally observed in PSP patients, intermediate values in PD patients, and lower values in ET. This observation remains indirect and should be confirmed in future studies combining inflammatory and neurodegenerative biomarkers.

The lack of a statistically significant difference in the NLR between the PD and control participants contrasts with previous studies reporting an increased NLR in PD patients [51]. Nevertheless, the direction of differences in the present cohort was broadly consistent with earlier findings, with the highest median NLR values observed in PSP patients and numerically higher values in PD patients than in controls. As the NLR primarily reflects neutrophil-related innate immune activity, its variability may depend on the systemic inflammatory status and may not consistently capture chronic, low-grade neuroinflammatory processes. Similarly, although the SII and AISI did not reach statistical significance, both indices were the highest in PSP patients. Because the SII and AISI integrate several hematological parameters involved in innate and adaptive immune responses, they may better reflect the cumulative burden of systemic inflammation than individual blood cell counts. In contrast, no clear between-group differences were observed for the SIRI, while the MLR values were comparable across groups, suggesting that monocyte-related immune alterations may be less prominent than platelet-related or neutrophil-related changes in the analyzed disorders.

The direction of the relationship between inflammation and neurodegeneration remains uncertain. Peripheral inflammatory alterations may contribute to neurodegenerative processes, but they may also arise as a consequence of ongoing neuronal injury, disease progression, disability, comorbidities, or systemic disease burden. Therefore, the observed differences in the PLR and other inflammatory indices should be interpreted as associations rather than evidence of causality.

None of the analyzed indices differed significantly between the groups, and the corresponding effect sizes were negligible to small. These findings do not demonstrate equivalence or exclude subtle biological differences; rather, they indicate that the present exploratory sample did not provide sufficient evidence of robust disorder-specific inflammatory profiles.

The nominally higher PLR observed in PSP patients compared with ET patients may represent a potentially relevant observation; however, this association did not remain statistically significant after a correction for multiple testing and should therefore be interpreted as exploratory. However, because the PLR is nonspecific, component-dependent, and influenced by multiple systemic factors, larger prospective studies combining inflammatory indices, individual blood cell counts, markers of platelet activation, detailed clinical phenotyping, and established neurodegenerative biomarkers, including NfL, are needed to clarify these relationships. The present cross-sectional study did not assess diagnostic accuracy, establish clinically applicable cutoff values, or evaluate changes over time. Accordingly, ROC analysis was not performed because the observed PLR difference was exploratory, did not remain significant after correction for multiple testing, and was not confirmed in the age- and sex-adjusted analysis. Moreover, deriving a cutoff value in this relatively small cohort without an independent validation group could produce an unstable estimate of diagnostic performance. Nevertheless, the absence of ROC analysis limits the assessment of the potential discriminatory performance and clinical utility of PLR. If validated in larger cohorts, PLR could potentially serve as an inexpensive complementary parameter within a multimodal assessment, particularly in settings with limited access to specialized biomarkers or advanced neuroimaging.

Although the diagnosis of PSP in our cohort was based on established clinical criteria rather than neuropathological confirmation, previous clinicopathological studies have demonstrated a high concordance between the final clinical diagnosis and neuropathological findings [52,53]. Therefore, while some degree of diagnostic misclassification remains possible, it is unlikely to have substantially affected the overall interpretation of our results.

## 5. Conclusions

In this exploratory cohort, preliminary differences in the peripheral inflammatory profiles were observed among the studied groups; however, these findings require confirmation in larger, independent cohorts. Among the analyzed blood-derived inflammatory indices, the PLR demonstrated the largest nominal between-group difference, with the highest values observed in patients with PSP and the lowest values in patients with ET. However, this association was not retained after a correction for multiple testing and an adjustment for potential confounders and should therefore be considered exploratory. Although the remaining indices did not reach statistical significance, the overall pattern of the results, including the higher median values of the NLR, SII, and AISI in PSP, may indicate a tendency toward greater peripheral immune activation in this disorder. These findings warrant further investigation in larger prospective studies. Future prospective, multicenter studies with larger cohorts, longitudinal follow-up, detailed medication data, disease-stage stratification, and standardized biomarker assessments are needed to clarify these findings.

## 6. Limitations

This study has several limitations. The data were collected from existing medical records, which may be subject to incomplete documentation and variability in laboratory assessments. The study included a relatively small sample size; thus, some potentially relevant associations may have remained undetected. Predefined exclusion criteria reduced the inclusion of participants with major clinically apparent inflammatory conditions, including infection, autoimmune or inflammatory disease, malignancy, hematological disorders, and immunosuppressive treatment, but could not eliminate residual confounding. Subclinical inflammation, medication use, smoking, body mass index, age, and sex may still have affected the results. The sensitivity analysis was limited by incomplete CRP and anti-inflammatory medication data. Furthermore, the patients included in this study differed with respect to the disease duration and time since diagnosis. Neuropathological confirmation was unavailable in the present study. Thus, the potential for diagnostic misclassification remains and could have diminished the observed between-group differences. Future prospective, multicenter studies with larger cohorts, longitudinal follow-up, detailed medication data, disease-stage stratification, and standardized biomarker assessments are needed to further clarify the presented findings.

## Figures and Tables

**Table 1:** Demographic characteristics of the study population.

Group	*n*	Mean Age ± SD [Years]	Median Age	Men *n* (%)	Women *n* (%)
ET	44	61.6 ± 14.3	65.5	24 (54.5%)	20 (45.5%)
PD	47	63.6 ± 10.7	63.0	22 (46.8%)	25 (53.2%)
PSP	44	67.3 ± 6.4	68.0	19 (43.2%)	25 (56.8%)
Control	45	63.3 ± 11.0	65.0	19 (42.2%)	26 (57.8%)

**Table 2:** Comparison of peripheral inflammatory markers in ET, PD, and PSP patients, and control participants.

Parameter	ET	PD	PSP	Control Participants	*p*-Value
NLR	1.95	2.04	2.21	1.9	0.524
	(1.47–2.63)	(1.55–2.74)	(1.62–2.95)	(1.49–2.44)	
PLR	112.03	121.37	142.93	118.75	0.045
	(88.29–141.37)	(95.54–150.84)	(112.17–183.75)	(92.64–145.61)	
MLR	0.296	0.265	0.265	0.264	0.898
	(0.221–0.392)	(0.201–0.354)	(0.214–0.373)	(0.198–0.333)	
SII	402.83	406.25	580.95	432	0.131
	(321.23–690.86)	(324.42–675.97)	(390.78–780.83)	(308.24–746.68)	
SIRI	1.05	0.99	1.03	0.94	0.675
	(0.73–1.41)	(0.71–1.33)	(0.76–1.48)	(0.69–1.27)	
AISI	243.66	197.5	286.45	223.89	0.168
	(152.87–403.16)	(132.49–326.38)	(181.72–471.90)	(145.74–351.41)	

Data are presented as the median (interquartile range, IQR). *p*-values correspond to the Kruskal–Wallis test.

## Data Availability

The data presented in this study are available from the corresponding author upon request. The data are not publicly available due to privacy and ethical restrictions.

## Supplementary Materials

The following supporting information can be downloaded at: https://www.mdpi.com/article/10.3390/diseases14080281/s1, Table S1: Absolute peripheral blood cell counts in ET, PD, and PSP patients, and control participants; Table S2: Effect-size estimates for comparisons of peripheral inflammatory indices; Table S3: Holm-adjusted *p*-values for comparisons of inflammatory indices; Table S4: Results of the sensitivity analysis restricted to participants with available CRP concentrations ≤ 5 mg/L. Figures S1–S6: Box-plot distributions of the NLR, PLR, MLR, SII, SIRI, and AISI, respectively, across the ET, PD, PSP, and control groups. Figure S2 additionally presents the exploratory post hoc comparison between the PSP and ET groups.

## Abbreviations

The following abbreviations are used in this manuscript:

AISI	aggregate index of systemic inflammation
CRP	C-reactive protein
CSF	cerebrospinal fluid
ET	essential tremor
IL-1β	interleukin-1 beta
IL-6	interleukin-6
IQR	interquartile range
MLR	monocyte-to-lymphocyte ratio
MSA-P	multiple system atrophy, parkinsonian type
NfL	neurofilament light chain
NLR	neutrophil-to-lymphocyte ratio
PD	Parkinson’s disease
PLR	platelet-to-lymphocyte ratio
PSP	progressive supranuclear palsy
PSP-RS	progressive supranuclear palsy-Richardson syndrome
SD	standard deviation
SII	systemic immune-inflammation index
SIRI	systemic inflammation response index
TNF-α	tumor necrosis factor alpha
