# Supplementary material for: Blood-Derived Inflammatory Indices Across Essential Tremor, Parkinson’s Disease, and Progressive Supranuclear Palsy: An Exploratory Retrospective Analysis
Source: Diseases. 2026 Aug 5;14(8):281. doi: 10.3390/diseases14080281 (PMC13512078; doi:10.3390/diseases14080281)
Supplement: Supplementary file 1 [file diseases-14-00281-s001.zip › Table S1.pdf]

Table S1. Absolute peripheral blood cell counts in ET, PD, and PSP patients, and control participants

	ET				PD				PSP				Control Participants			
Variable	Median	Min	Max	IQR	Median	Min	Max	IQR	Median	Min	Max	IQR	Median	Min	Max	IQR
Lymphocytes	1.885	0.97	3.26	0.840	1.820	0.49	3.59	0.870	1.775	0.94	4.33	0.710	1.740	0.70	3.44	0.980
Neutrophils	3.700	1.91	6.88	1.635	3.550	1.66	7.39	2.000	4.005	1.31	10.00	1.890	3.720	1.87	9.50	1.950
Platelets	228.500	96.00	412.00	73.500	210.000	110.00	358.00	70.000	242.000	147.00	456.00	78.000	237.000	113.00	348.00	65.000
Monocytes	0.545	0.24	0.92	0.260	0.490	0.20	0.84	0.280	0.485	0.27	0.85	0.205	0.500	0.10	1.04	0.270
