# Supplementary material for: Blood-Derived Inflammatory Indices Across Essential Tremor, Parkinson’s Disease, and Progressive Supranuclear Palsy: An Exploratory Retrospective Analysis
Source: Diseases. 2026 Aug 5;14(8):281. doi: 10.3390/diseases14080281 (PMC13512078; doi:10.3390/diseases14080281)
Supplement: Supplementary file 1 [file diseases-14-00281-s001.zip › Table S2.pdf]

Table S2. Effect-size estimates for comparisons of peripheral inflammatory indices.

Marker	Kruskal–Wallis H	p-value	ε^2
NLR	2.247	0.523	0.000
PLR	8.064	0.045	0.029
MLR	0.596	0.897	0.000
SII	5.627	0.131	0.015
SIRI	1.533	0.675	0.000
AISI	5.048	0.168	0.012

For the PSP versus ET comparison, Cliff's delta for PLR was ***0.336*** *(small-to-moderate effect).*
