# Supplementary material for: Blood-Derived Inflammatory Indices Across Essential Tremor, Parkinson’s Disease, and Progressive Supranuclear Palsy: An Exploratory Retrospective Analysis
Source: Diseases. 2026 Aug 5;14(8):281. doi: 10.3390/diseases14080281 (PMC13512078; doi:10.3390/diseases14080281)
Supplement: Supplementary file 1 [file diseases-14-00281-s001.zip › Table S3.pdf]

Table S3. Holm-adjusted *p*-values for comparisons of inflammatory indices.

**Marker**	**Kruskal–Wallis p**	**Holm-adjusted p**
NLR	0.524	1.000
PLR	0.045	0.268
MLR	0.898	1.000
SII	0.131	0.656
SIRI	0.675	1.000
AISI	0.168	0.673
