# Supplementary material for: Blood-Derived Inflammatory Indices Across Essential Tremor, Parkinson’s Disease, and Progressive Supranuclear Palsy: An Exploratory Retrospective Analysis
Source: Diseases. 2026 Aug 5;14(8):281. doi: 10.3390/diseases14080281 (PMC13512078; doi:10.3390/diseases14080281)
Supplement: Supplementary file 1 [file diseases-14-00281-s001.zip › Table S4.pdf]

Supplementary Table S4. Results of the sensitivity analysis restricted to participants with available C-reactive protein concentrations ≤5 mg/L.

**Marker**	**Kruskal–Wallis H**	**p-value**	**ε^2**
NLR	2.243	0.523	0.000
PLR	3.612	0.307	0.006
MLR	1.688	0.640	0.000
SII	4.451	0.217	0.015
SIRI	3.564	0.313	0.006
AISI	5.575	0.134	0.027

The sensitivity analysis included 101 participants (ET, n = 19; PD, n = 35; PSP, n = 23; control participants, n = 24). Effect size for the Kruskal–Wallis test was estimated using epsilon-squared (ε^2).
